# Export of dietary lipids via emergent insects from eutrophic fishponds

**DOI:** 10.1007/s10750-022-05040-2

**Published:** 2022-10-27

**Authors:** Lena Fehlinger, Margaux Mathieu-Resuge, Matthias Pilecky, Tarn Preet Parmar, Cornelia W. Twining, Dominik Martin-Creuzburg, Martin J. Kainz

**Affiliations:** 1 WasserCluster Lunz - Biologische Station GmbH; Inter-university Center for Aquatic Ecosystem Research, Dr. Carl Kupelwieser Promenade 5, 3293 Lunz am See, Austria; 2 University for Continuing Education Krems, Dr.-Karl-Dorrek-Straße 30, 3500 Krems, Austria; 3 Department of Biology, Limnological Institute, University of Konstanz, Mainaustraße 252, 78464 Konstanz, Germany; 4 Department of Fish Ecology and Evolution, EAWAG: Swiss Federal Institute of Aquatic Sciences and Technology, Seestrasse 79, 6047 Kastanienbaum, Switzerland; 5 Department of Aquatic Ecology, Research Station Bad Saarow, BTU Cottbus-Senftenberg, Seestraße 45, 15526 Bad Saarow, Germany

**Keywords:** Dietary energy, Eutrophic, Fishpond, Aquatic insects, Emergence, Ecosystem services

## Abstract

Fishponds, despite being globally abundant, have mainly been considered as food production sites and have received little scientific attention in terms of their ecological contributions to the surrounding terrestrial environment. Emergent insects from fishponds may be important contributors of lipids and essential fatty acids to terrestrial ecosystems. In this field study, we investigated nine eutrophic fishponds in Austria from June to September 2020 to examine how Chlorophyll-*a* concentrations affect the biomass of emergent insect taxa (i.e., quantity of dietary subsidies; *n* = 108) and their total lipid and long-chain polyunsaturated fatty acid content (LC-PUFA, i.e., quality of dietary subsidies; *n* = 94). Chironomidae and Chaoboridae were the most abundant emergent insect taxa, followed by Trichoptera, Ephemeroptera, and Odonata. A total of 1068 kg of emergent insect dry mass were exported from these ponds (65.3 hectares). Chironomidae alone exported 103 kg of total lipids and 9.4 kg of omega-3 PUFA. Increasing Chl-*a* concentrations were associated with decreasing biomass export and a decrease in total lipid and LC-PUFA export via emergent Chironomidae. The PUFA composition of emergent insect taxa differed significantly from dietary algae, suggesting selective PUFA retention by insects. The export of insect biomass from these eutrophic carp ponds was higher than that previously reported from oligotrophic lakes. However, lower biomass and diversity are exported from the fishponds compared to managed ponds. Nonetheless, our data suggest that fishponds provide crucial ecosystem services to terrestrial consumers by contributing essential dietary nutrients to consumer diets via emergent insects.

**Supplementary Information:**

The online version contains supplementary material available at 10.1007/s10750-022-05040-2.

## Introduction

Emergent insects play a crucial role for riparian food webs as sources of dietary energy from aquatic ecosystems, subsidizing a diversity of consumers including insectivorous birds (Twining et al., 2016, 2018), spiders (Kowarik et al., 2021; Mathieu-Resuge et al., 2021a; Twining et al., 2021), and bats (Henschel et al., 2001; Kato et al., 2003; Fukui et al., 2006; Lam et al., 2013). Although the trophic transfer of energy via emergent insects from lotic ecosystems is well documented (Nakano & Murakami, 2001), only more recently have studies focused on the dietary contribution of emergent insects from lakes to terrestrial consumers (e.g., Gratton & Vander Zanden, 2009; Martin-Creuzburg et al., 2017; Mathieu-Resuge et al., 2021a, b). For example, up to 4 g m^−2^ year^−1^ of insect dry mass emerged from a shallow lake in Iceland (Dreyer et al., 2015), and up to 1 g m^−2^ year^−1^ were exported from temporary ponds to terrestrial ecosystems (Fritz & Whiles, 2021). Factors such as pond productivity (Scharnweber et al., 2019), food availability, temperature, and light (Ivković et al., 2013; Raitif et al., 2018), as well as the presence of benthivorous fish (Wesner, 2016) can lead to high variability in aquatic insect emergence. In addition, the emergence of aquatic insects also fluctuates throughout the year as a result of seasonal shifts in shading and temperature (Nakano & Murakami 2001). Consequently, there is still an urgent need to quantify insect emergence throughout the year across a diversity of aquatic ecosystems.

In the current era of rapidly declining biodiversity, calls to conserve and manage ponds at the ecosystem scale have become louder, especially since ponds provide valuable habitats for many species, including emergent insects (Oertli et al., 2002). In a study conducted on farm ponds, Lewis-Phillips et al. (2020) found a 25-fold increase of emergent insect biomass in managed versus unmanaged ponds which was highly linked to open, unshaded water surface area. Further, there are indications that pond networks provide increased insect biomass supply for terrestrial consumers (Berzins et al., 2021). In landscapes containing clusters of managed ponds, the presence of various stages of pond succession leads to more diverse aquatic communities, which are available as a food resource for both aquatic and terrestrial consumers (Lewis-Phillips et al., 2020).

Although there are studies focusing on emergent insect biomass, there is little knowledge about the trophic transfer of lipids via emergent insects from ponds. The number of ponds worldwide has been estimated to exceed 300 million (Downing & Duarte, 2009). However, those numbers are subject to change (Pekel et al., 2016); e.g., in some European countries, over 90% of ponds were already lost (EPCN, 2008). The export of dietary energy and essential lipids from ponds via emergent insects may be an extremely important, underestimated and little-acknowledged ecosystem service provided by millions of increasingly threatened ponds across the European landscape (Oertli et al., 2009).

Emergent insects acquire dietary lipids and essential fatty acids (FA) during their larval life stage. Dietary supply of long-chain (> C18) polyunsaturated fatty acids (LC-PUFA) is crucial for aquatic (Von Elert 2002; Ebm et al., 2021; Vesterinen et al., 2021) as well as terrestrial consumers (Twining et al., 2016, 2018; Mathieu-Resuge et al., 2021b). Some algal taxa, such as diatoms, contain high levels of n-3 LC-PUFA, including eicosapentaenoic acid (EPA; Gladyshev et al., 2013), which can be transferred to aquatic insect larvae (Guo et al., 2016). In addition to dietary energy, consumers also depend upon specific dietary compounds like PUFA to support their somatic growth, reproduction, and survival (Sabo & Power, 2002; Burdon & Harding, 2008; Martin-Creuzburg et al. 2010; Fritz et al., 2017). For example, bats can go into longer and undisturbed torpor if their autumn-diet was rich in PUFA, which renders them less susceptible to infections (Frank et al., 2012). Tree swallow chicks feeding on an n-3 LC-PUFA-rich diet had better condition, faster growth, and enhanced immune function compared to those feeding on a diet with more of the shorter-chain PUFA (Twining et al., 2016) and those with access to higher aquatic insect biomass during their nestling phase were more likely to survive (Twining et al., 2018). A positive impact on the immune system was also reported for stream riparian wolf spiders that fed on emergent insects (Fritz et al., 2017). Thus, the dietary provision of LC-PUFA from emergent insects may support the fitness of terrestrial consumers, highlighting the ecological importance of dietary LC-PUFA transfer from aquatic to terrestrial ecosystems.

Emergent insects vary in their LC-PUFA content and composition (Martin-Creuzburg et al., 2017; Mathieu-Resuge et al., 2021b; Twining et al., 2021). For example, Chaoboridae contain substantially higher contents of EPA (~ 25 mg g dw^−1^) and docosahexaenoic acid (DHA; ~ 5 mg g dw^−1^) than Chironomidae and Odonata (~ 5 mg EPA g dw^−1^ and only traces of DHA; Gladyshev et al., 2009; Martin-Creuzburg et al., 2017; Popova et al., 2017; Twining et al., 2021). In addition to taxonomic differences, the trophic condition of water bodies can also affect the quantity of LC-PUFA retained in aquatic insects (Scharnweber et al. 2019). Eutrophication can cause a shift from diatom-dominated assemblages to communities primarily consisting of cyanobacteria that lack LC-PUFA (Rasconi et al., 2015, 2017). Hence, the dietary quality of seston and/or periphyton consumed by emergent insects may decrease with eutrophication (Smith, 2003; Taipale et al., 2013; Strandberg et al., 2015).

Fishponds provide millions of tons of fish for human consumption per year worldwide, in particular carp (Steffens & Wirth, 2007; FAO, 2012; Böhm et al., 2014; Rahman, 2015; Roy et al., 2020) and tilapia (Norman-Lopez & Bjørndal, 2009), which are among the most consumed fish species (FAO, 2017). Fishponds that are heavily stocked tend to support less diverse macroinvertebrate communities than ponds with fewer fish (Kloskowski, 2011). Increased turbidity (e.g., bioturbation of carp, sediment resuspension; see in review by Weber & Brown, 2009) and lower oxygen contents favor generalist taxa like Chironomidae rather than more sensitive taxa (Lewis-Phillips et al., 2020). In intensively used carp ponds, macroinvertebrate abundances are expected to be drastically reduced compared to natural ponds. However, in moderately stocked ponds with macrophyte areas, macrophyte zones can act as a refuge in which emergent insect production is high (Kajgrova et al., 2021). Thus, depending on their management, carp ponds may be locally important areas of insect emergence, providing valuable dietary energy and essential nutrients to riparian consumers (Gratton & Vander Zanden, 2009; Dreyer et al., 2015).

The current lack of knowledge about the role of fishponds in providing dietary energy and lipids via emergent insects to consumers motivated us to conduct a field study to investigate how the trophic state of fishponds affects (i) the export of emergent insect biomass to terrestrial ecosystems especially throughout the summer season, and (ii) lipid and fatty acid contents of both algae and emergent insects. We hypothesized that seston Chl-a concentrations of fishponds are positively correlated with total insect biomass export, but negatively with LC-PUFA export, due to a higher share of LC-PUFA-deficient algae in nutrient-rich ponds. To our knowledge, this is the first study that quantifies the food quantity and quality, as assessed by total lipid and PUFA contents, exported by emergent insects from carp ponds.

## Material and methods

### Field sampling

Nine carp ponds with an average depth of 1.5 m were sampled in northern Austria (Fig. 1.1 A, Appendix 1), around the city of Waidhofen an der Thaya (48.8155°N, 15.2833°E) (Fig. 1.1 B, Appendix 1). These study ponds, i.e., the largest pond Jägerteich (JAG; 38.1 ha), Kiebitzteich (KIEB; 2.5 ha), Furtteich (FURT; 0.7 ha), Dachetteich (DACH; 3 ha), Stadtteich (STADT; 3.9 ha), Engelbrechtsteich (ENG; 3 ha), Gerhartsteich (GER; 3.4 ha), Herrenteich (HERR; 10.1 ha), and Dürnhofteich (DURN; 0.6 ha), contained patchy littoral vegetation (mostly reeds) and were surrounded by forests and agriculture. The climate in the region is continentally influenced with cold nights even in summer and annual temperature averages around 6–7 °C (source: KLAR! Waldviertler Kernland), which is why it takes carp, which have a temperature optimum around 25 °C, around 3 to 4 summers to fully mature (Bauer, 2014). The study ponds are used for commercial reasons (carp production), except DURN that has not been stocked with fish for years. Generally, in the Waldviertel region, ponds have not been subjected to mineral fertilizers since the 1980s and ponds are only moderately stocked (300–600 fish/ha; Bauer, 2014). The fish biomass among the study ponds differed: DURN did not contain any stocked fish, FURT contained only fish larvae, STADT, KIEB, DACH, and ENG contained one summer old carp, JAG and GER contained two summers old carp, and HERR contained three summers old carp. Fish abundance ranged from 4500 (DACH) to 24,000 (JAG) individuals.

### Sample collection

Emergent insects and seston samples were collected in triplicate from each of the study ponds, weekly in June and twice a month during the rest of the sampling season. Three emergence traps (surface area: 0.36 m^2^, covered with a 500 µm net; see Martin-Creuzburg et al., 2017) per pond were deployed in June 2020. White collection containers installed on top of the pyramid-shaped trap facilitated the sampling of the insects with a self-made aspirator. They were placed so as to cover the deeper zones of each of the ponds and the shoreline, under overhanging branches or near the reeds to include potential heterogeneity in the substrate. The number of sampled ponds (*n* = 9) and the size of emergence traps have to be considered especially in regard to the extrapolations of emergent insect biomass, even though the sampling design was adjusted to explicitly account for substrate heterogeneity and potential variations in emergence. Emergent insects were collected for biomass estimation, total lipid, and fatty acid analyses. Insects were stored at − 80 °C, freeze dried (Virtis Genesis Freeze dryer) for 24 h, identified to family or genus level, and stored at − 20 °C until further biochemical analysis.

Seston samples were collected using a Schindler trap (UWITEC™, Mondsee, Austria) and pre-filtered through a 30-µm mesh and then filtered onto pre-weighed, pre-combusted GF/C filters (VWR™, 47 mm diameter, ~ 1.2 µm pore size), which were subsequently frozen at − 80 °C, freeze dried for 24 h, weighed again, and stored at − 20 °C until further processing for lipid analysis. Freeze-dried seston was assessed as mass fraction per unit biomass (i.e., mg per g dry weight^−1^; dw).

Physical and chemical parameters, including temperature (°C), pH, specific conductivity (mS^−1^ cm^−1^) of pond water, were recorded once a month from June to September using a Hydrolab HL7 multiparameter-probe (Ott Hydromet). Triplicates of filtered pond water (Supor® Membrane Filters, 0.2 µm pore size; Pall Corporation) were used for analysis of dissolved organic carbon (DOC; Shimadzu Total Organic Carbon analyzer, TOC-L series).

For chlorophyll-*a* (Chl-*a*) measurements, pond water (3 l) was filtered in the dark on GF/C filters (VWR™), extracted using acetone (Arar & Collins, 1997) and subsequently measured on a spectrofluorometer (Hitachi F-7000).

### Biomass quantification

The biomass of emergent insects (EI_biom_) per meter square (m^2^) was calculated as

1 $${\mathrm{EI}}_{\mathrm{biom}}=\frac{\mathrm{Insect biomass}}{0.36}$$

where Insect biomass corresponds to the biomass of each trap (mg^−1^ dw^−1^), and 0.36 is the trap area in m^2^. To enable comparison among ponds and also with already existing biomass values in the literature (e.g., Mathieu-Resuge et al., 2021b), the values of biomass per square meter were used for further analysis (Kruskal–Wallis; KW test) to test for differences among ponds and months.

For extrapolation purposes, to get an estimate of the magnitude of the insect flux to the adjacent terrestrial ecosystem, the three replicates EI_biom_ obtained from each of the study ponds were averaged to obtain the mean EI_biom_ per m^2^, i.e., ${\mathrm{EI m}2}_{\mathrm{biom}}$.

The daily emerged insect biomass exported per m^2^ (*EI*_*d*_) was assessed as

2 $${\mathrm{EI}}_{d}=\frac{{\mathrm{EI m}2}_{\mathrm{biom}}}{n \mathrm{day}},$$

where *n* day corresponds to the number of days between the sampling events.

The daily emerged insect biomass per pond (EI_*p*_) was assessed as

3 $${\text{EI}}_{p} = EI_{d} *Ps$$

where *Ps* represents the pond surface in square meters.

The estimation of the monthly emerged insects biomass (EI_*m*_) exported per pond was assessed as

4 $${\mathrm{EI}}_{m}=\mathrm{EI}p*x$$

where *x* corresponds to the number of days per month.

The extrapolated biomass values for each month were summed to obtain the overall extrapolated biomass for the entire sampling period (EI_S_).

5 $${\text{EI}}_{{\text{s}}} = {\text{EI}}_{{{\text{m1}}}} + {\text{EI}}_{{{\text{m2}}}} + {\text{EI}}_{{{\text{m3}}}} + {\text{EI}}_{{{\text{m4}}}}$$

where EI_m1_ represents the extrapolated monthly biomass for June, EI_m2_ for July, EI_m3_ for August, and EI_m4_ for September. Biomass values were first calculated in milligrams (mg) and then converted to kilogram (kg) values.

To calculate the extrapolated total lipids (TL) and omega-3 (n-3) FA export, the obtained TL and n-3 values (mg g dw^−1^) from the individual samples were averaged to get one mean value for each pond in each month of the sampling season (TL_mean_ and n-3_mean_). To calculate TL and n-3 export (TL_exp_ and n-3_exp_) via emergent insects from each pond per month, we used

6 $${\text{TL}}_{{{\text{exp}}}} = {\text{SEI}}_{{\text{m}}} *{\text{STL}}_{{{\text{mean}}}}$$

7 $${\text{n - 3}}_{{{\text{exp}}}} = {\text{ SEI}}_{m} *{\text{ Sn - 3}}_{{{\text{mean}}}}$$

where SEI_*m*_ represents the extrapolated insect biomass for all ponds, and STL_mean_ and Sn-3_mean_ are the overall extrapolated contents of TL and n-3 exported via emergent insects for the whole sampling season reported in grams (g).

### Lipid and fatty acid analyses

Lipids were extracted as described by Heissenberger et al. (2010). Freeze-dried emergent insects and filters (i.e., seston) were mixed with chloroform:methanol (2:1, vol/vol) followed by sonication, vortexing, and centrifuging three times to remove non-lipid materials. Pooled organic phases were evaporated to a final volume of 1.5 ml under N_2_. Total lipid contents were obtained by injecting 100 µl of the extract into pre-weighed tin capsules, which were weighed again after the liquid extract had evaporated. For fatty acid methyl esters (FAME) formation, a known volume of lipid extracts was incubated with sulfuric acid:methanol (1:100 vol/vol) for 16 h at 50 °C, following the addition of KHCO_3_ and hexane. Samples were shaken, vortexed, and centrifuged, and the upper organic layers collected 2 times, pooled, and concentrated under N_2_.

FAME were analyzed using a gas chromatograph (TRACE GC THERMO, Detector: FID 260 °C, Carrier gas: He: 1 ml^−1^ min^−1^, Detector gases: H_2_: 40 ml^−1^ min^−1^, N_2_: 45 ml^−1^ min^−1^, air: 450 ml^−1^ min^−1^, temperature ramp: 140 °C (5 min)–4 °C min^−1^–240 °C (20 min) = 50 min) equipped with a temperature-programmable injector and an autosampler. A Supelco SP-2560 column (100 m, 25 mm i.d., 0.2 µm film thickness) was used for FAME separation. Comparison of the retention times with standards led to identification of FAME (37-component FAME Mix, 47,885-U, Supelco; Sigma-Aldrich, Bellefonte, Pennsylvania). Chromeleon 7™ was used for peak integration and concentrations of FA were calculated based on individual calibration curves. Results were expressed as mass fractions (e.g., mg FAME g dw^−1^) or reported as mass percentages (%).

### Data analysis

Data were analyzed and visualized using R (R 4.1.2; R. Core Team, 2021) using the packages *vegan, ggplot2, leaflet, PMCMR, reshape, multcompView, nlme, and conover.test.* The significance level *P* < 0.05 was applied for all tests. After checking the normality (Shapiro–Wilk test) and homoscedasticity (Levene test) of PUFA data distributions, PERMANOVA was applied to compare lipid and fatty acid contents and to test the interaction among variables (i.e., for Chironomidae and seston, respectively, among ponds and months). Because interactions were found among the months and ponds, we separately analyzed the changes in PUFA composition using non-parametric KW tests with Conover post hoc analysis (utilizing Holm-Bonferroni correction for multiple testing as adjustment method) for each variable. We applied KW tests to test for differences in the selected n-3 PUFA and n-3:n-6 ratio among the ponds and the sampling months in Chironomidae and seston, as well as for the comparison of biomass changes among months and ponds. Principal component analysis (PCA) was used to visualize differences in FA composition between Chironomidae and seston. PCA was performed using log-transformed FA data (%), and only FA > 1.5% were considered. Linear mixed effect (LME) models were conducted using the lme() function to test the predictive power of Chl-*a* on the export of insect biomass or lipid and n-3 PUFA contents when considering ponds as random factor. For the LME models, log-transformed values of insect biomass, chironomid biomass, seston biomass, total lipids, and n-3 PUFA contents of Chironomidae were used. Linear models were used to visualize the relationship between total insect biomass extrapolated in kilograms (kg), n-3 PUFA content of Chironomidae (mg/g), total lipid content of Chironomidae (mg^−1^ g^−1^), and Chlorophyll-*a* content (µg^−1^ l^−1^). Log-transformed values were used to establish the linear models.

## Results

### Physical and chemical parameters of ponds

In June, Chl-*a* concentrations of most ponds were low (1–4 μg l^−1^), except in the ponds HERR, JAG, and ENG (7–12 μg l^−1^; Table 2.1, Appendix 2). Chl*-a* steadily increased in July and August, and were highest in September (ranging from 55 to 140 μg l^−1^; Table 2.1, Appendix 2). The water temperature of the ponds increased from 16 to 18 °C in June to 23–27 °C in August and then decreased in September (16–18 °C; Table 2.1, Appendix 2). The ponds were circumneutral to alkaline (pH: 6.8–9.4; Table 2.1, Appendix 2) and the water conductivity remained similar from June (140–630 μS cm^−1^) to September (130–460 μS cm^−1^; Table 2.1, Appendix 2). The DOC concentrations of the ponds ranged from 8 to 16 mg l^−1^ throughout the study period (Table 2.1, Appendix 2).

### Biomass and lipids of seston and emerged Chironomidae

The standing stock of the most readily edible algal size fraction, commonly referred to as edible seston (algae < 30 µm; Burns, 1968) was significantly higher in June (ranging from 4.4 to 12.1 mg g dw^−1^) and September (from 4.6 to 8.1 mg g dw^−1^) compared to July (from 3.4 to 7 mg g dw^−1^) and August (from 2.6 to 5.5 mg g dw^−1^; KW; χ^2^ = 14.8, df = 3, *P* = 0.002).

The extrapolated biomass of emerged insects from all nine study ponds was 1068 kg dw for the entire sampling period (June–September 2020; Table 3.1, Appendix 3). The sampled ponds had a total surface of 65.3 ha, which equated to approximately 16.4 kg dw of total insect biomass per ha or 1.64 g dw per m^2^ in the four months of sampling. Samples of biomass were taken every two weeks throughout the sampling season.

**Fig. 1:**
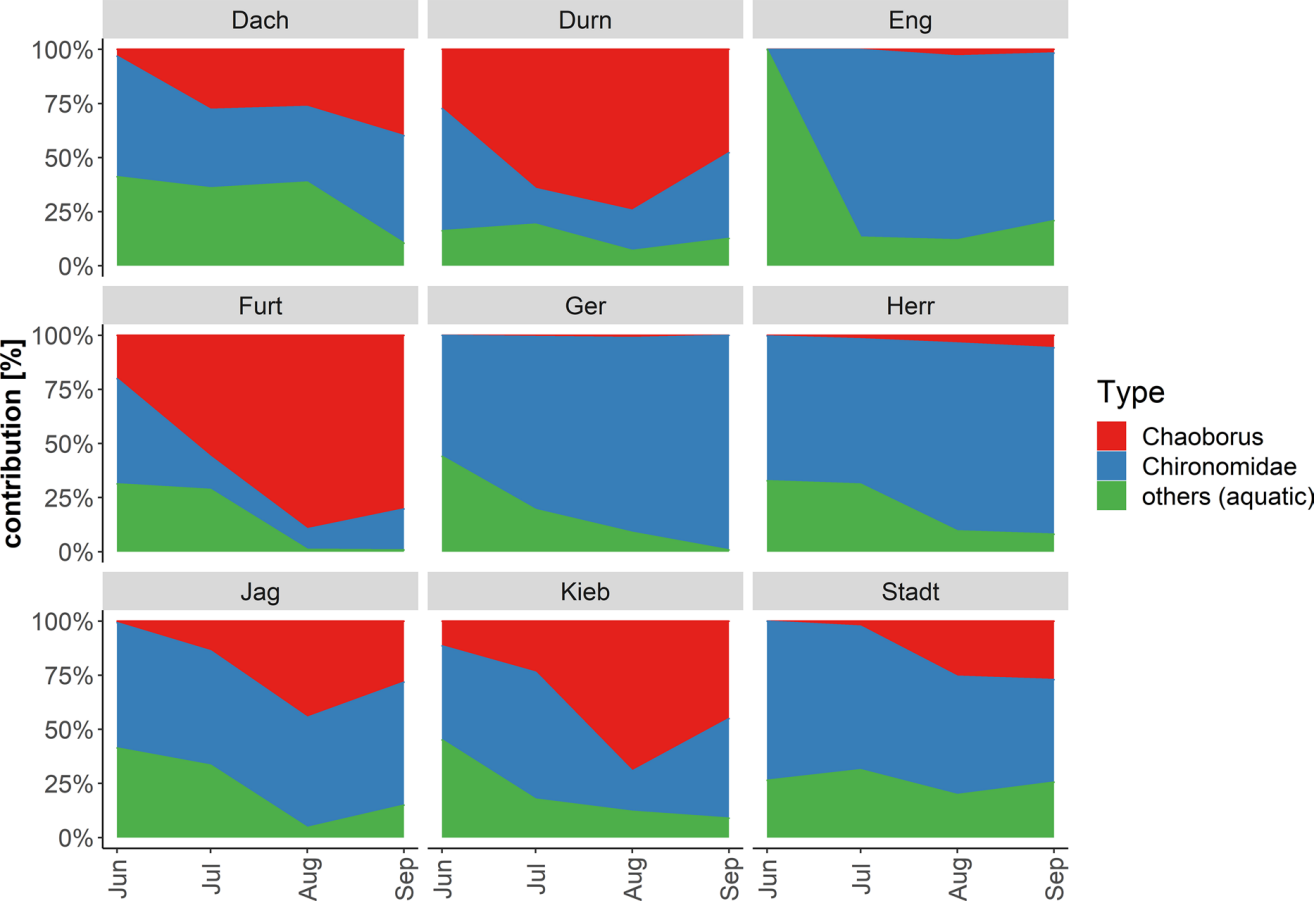
Relative contribution (in %) of Chironomidae, Chaoboridae, and other aquatic insects (i.e., Odonata, mainly Coenagrionidae; Diptera (Ceratopogonidae, Culicidae); Ephemeroptera, and Trichoptera) to the total biomass emerging from the nine ponds throughout the sampling period (June–September 2020)

The majority of the insect biomass from the traps of all ponds consisted of Chironomidae and Chaoboridae (Fig. 1, Table 3.1, Appendix 3). During the entire sampling period, the extrapolated biomass of emerged Chironomidae and Chaoboridae varied considerably among ponds and was the highest at the largest pond; i.e., JAG (482 kg and 72 kg, respectively). FURT and DURN exported the lowest Chironomidae biomass (2.9 kg and 2.5 kg; Table 3.1, Appendix 3), and exported a similar flux of Chaoboridae biomass (2.7 kg and 2.9 kg). In all other ponds, Chironomidae contributed far more to the overall biomass than Chaoboridae (Table 3.1, Appendix 3). The lowest Chaoboridae biomass was exported from GER (0.1 kg) and ENG (0.6 kg).

Other emergent insect taxa collected in the traps were Odonata (mainly Coenagrionidae), other Diptera (Ceratopogonidae, Culicidae, Ephydridae), Ephemeroptera, and Trichoptera. These groups were not processed for FA analysis due to low biomass, but were considered for biomass export calculations. All taxa except Chironomidae and Chaoboridae were only found in single traps, dispersed over the ponds and not in every month, often only single individuals with not enough biomass for FA analysis (< 2 mg dw; Table 3.2, Appendix 3). A few samples of Odonata (*n* = 10) and Chaoboridae (*n* = 12) were used for lipid and FA analysis, but due to the low sample size, they were not considered for statistical analysis. Odonata were only recorded from the ponds KIEB (*n* = 7) and FURT (*n* = 1) in June and from STADT in August (*n* = 1) and September (*n* = 1). In July, Chaoboridae were found in all ponds except in ENG and HERR. In August, Chaoboridae were found in all ponds, but in ENG, GER, and HERR, the biomass was too low for FA analysis (< 2 mg dw; *n* = 6). Taxa that were not found throughout the summer months or not in all ponds were not included because the seasonal effect could not be compared and the lipid exports could not be quantified as for the more prevalent taxa.

Overall, the total insect biomass per square meter (Table 3.2, Appendix 3) did not significantly differ among months (KW; χ^2^ = 0.77, df = 3, *P* = 0.86) or among ponds (KW; χ^2^ = 4.5, df = 8, *P* = 0.81). Similarly, the exported Chironomidae biomass per square meter (Table 3.2, Appendix 3) did not significantly differ among months (KW; χ^2^ = 1.31, df = 3, *P* = 0.73) or among ponds (KW; χ^2^ = 13.28, df = 8, *P* = 0.10).

No significant differences in Chaoboridae biomass per square meter (Table 3.2, Appendix 3) were found between months (KW; χ^2^ = 5.31, df = 3, *P* = 0.15), but there were significant differences among ponds (KW; χ^2^ = 25.70, df = 8, *P* = 0.001). Differences were found between DURN compared with ENG (*P* < 0.001), GER (*P* < 0.001) and HERR (*P* < 0.01), between FURT compared with ENG (*P* < 0.001), GER (*P* < 0.001) and HERR (*P* < 0.01) and between KIEB compared with ENG (*P* = 0.002), GER (*P* < 0.001), and HERR (*P* < 0.01). Chaoboridae biomass per square meter in DURN, FURT, and KIEB were higher throughout the months than in ENG, GER, and HERR (Table 3.2, Appendix 3).

The total lipids exported (calculated from extrapolated biomass) via Chironomidae, Chaoboridae, and Odonata from all studied ponds during the study period was 110 kg, of which Chironomidae contributed 102.8 kg (Table 3.3, Appendix 3; Fig. 3). Similarly, a total of 10.7 kg n-3 PUFA were exported via those taxa, of which Chironomidae exported most of these n-3 PUFA (9.4 kg) from all study ponds (Table 3.3, Appendix 3; Fig. 3). In terms of total lipids, this is equivalent to around 1.7 kg of total lipids per ha or 0.17 g per m^2^ exported via Chironomidae, Chaoboridae, and Odonata during the study period (4 months).

### Omega-3 PUFA contents in seston and Chironomidae

The FA composition of seston differed significantly among months (PERMANOVA, *F* = 5.90, df = 3, *R*^2^ = 0.15, *P* < 0.05) and ponds (PERMANOVA, *F* = 6.13, df = 8, *R*^2^ = 0.33, *P* < 0.05), with seston containing less linoleic acid (LIN, 18:2n-6; between 1 and 13%), but more DHA (between 0 and 6%) than Chironomidae. The FA composition of Chironomidae differed significantly among months (PERMANOVA, *F* = 3.05, df = 3, *R*^2^ = 0.10, *P* < 0.01) and ponds (PERMANOVA, *F* = 1.84, df = 8, *R*^2^ = 0.16, *P* < 0.01), with Chironomidae containing more LIN (between 4 and 28%) and less DHA (between 0 and 4%) than seston.

For both emergent Chironomidae and seston FA profiles, a significant interaction was detected between month and pond (PERMANOVA, Chironomidae Month*Pond, *F* = 2.08, df = 24, *R*^2^ = 0.37, *P* < 0.05; Seston Month*Pond, *F* = 15.71, df = 24, *R*^2^ = 0.44, *P* < 0.05).

The DHA contents of seston differed significantly among months (KW; χ^2^ = 36.9, df = 3, *P* < 0.005), except between July and August (*P* = 0.41). The EPA content also varied significantly among months (KW; χ^2^ = 29.9, df = 3, *P* < 0.005), i.e., between June and July (*P* = 0.0005), June and September (*P* = 0.0001), and August and September (*P* = 0.0002). The ALA content in seston varied significantly among months (KW; χ^2^ = 22.6, df = 3, *P* < 0.005), except between June and July (*P* = 0.16) and between July and August (*P* = 0.11). The n-3:n-6 PUFA ratio in seston differed significantly among all months (KW; χ^2^ = 39.1, df = 3, *P* < 0.005).

The content of the following PUFA in seston differed significantly among ponds (Table 3.4, Appendix 3): DHA (KW; χ^2^ = 35.95, df = 8, *P* < 0.005), EPA (KW; χ^2^ = 37.2, df = 8, *P* < 0.005), and ALA (KW; χ^2^ = 55.8, df = 8, *P* < 0.005). The n-3:n-6 PUFA ratio in seston did not differ significantly among the ponds (KW; χ^2^ = 16.26, df = 8, *P* = 0.04, Table 3.4, Appendix 3).

**Fig. 2:**
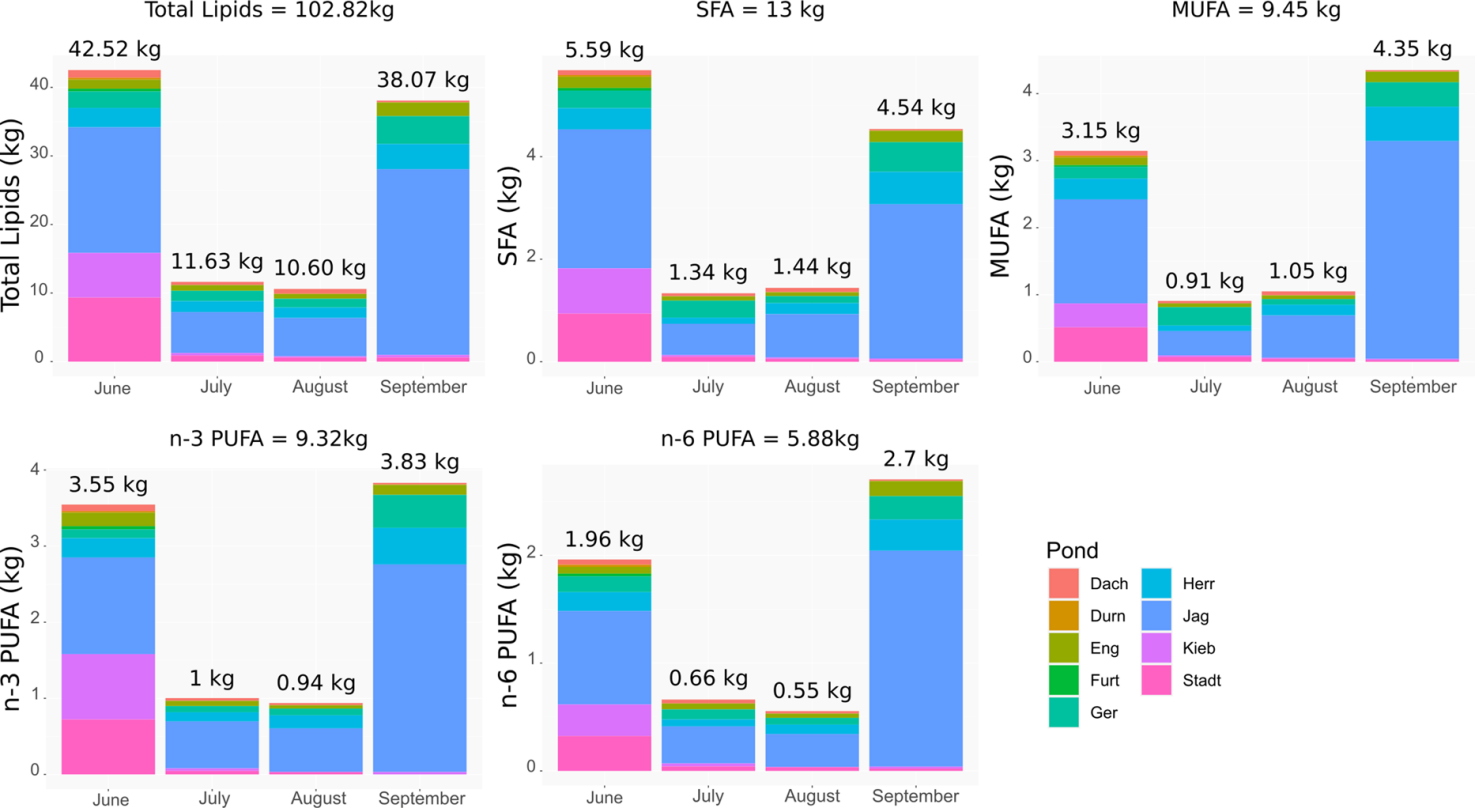
Total export in kilograms (kg) in the four months of sampling via Chironomidae. *MUFA* monounsaturated fatty acids, *n-3 PUFA* omega-3 polyunsaturated fatty acids, *n-6 PUFA* omega-6 polyunsaturated fatty acids, *SFA* saturated fatty acids. The nine sampled ponds are represented by randomly chosen colors. Fish ponds: *JAG* Jägerteich, *KIEB* Kiebitzteich, *FURT* Furtteich, *DACH* Dachetteich, *STADT* Stadtteich, *HERR* Herrenteich, *ENG* Engelbrechtsteich, *GER* Gerhartsteich, *DURN* Dürnhofteich

The export of total lipids via Chironomidae amounted to 102.8 kg, of which 9.3 kg were n-3 PUFA and 5.9 kg were n-6 PUFA (Fig. 2). Chironomidae also contained 9.5 kg of monounsaturated fatty acids (MUFA; Fig. 2) and 13 kg of saturated fatty acids (SAFA; Fig. 2). In June and September, the export of Chironomidae was generally higher than in July and August (Fig. 2).

The DHA and ALA content of Chironomidae and the n-3:n-6 PUFA ratio in Chironomidae did not differ significantly among months (KW; DHA: χ^2^ = 8.95, df = 3, *P* = 0.03; ALA: χ^2^ = 2.31, df = 3, *P* = 0.51; n-3:n-6 PUFA ratio: χ^2^ = 7.72, df = 3, *P* = 0.05; Table 3.5, Appendix 3). The EPA content of Chironomidae differed significantly between the months of June and July (KW; χ^2^ = 17.82, df = 3, *P* = 0.01) and between June and August (*P* < 0.001).

The EPA and ALA contents and the n-3:n-6 PUFA ratio in Chironomidae did not significantly differ among ponds (KW; EPA: χ^2^ = 4.6, df = 8, *P* = 0.8; ALA: χ^2^ = 13.11, df = 8, *P* = 0.11; n-3:n-6 ratio: χ^2^ = 9.68, df = 8, *P* = 0.29; Table 3.5, Appendix 3). However, DHA in Chironomidae from the ponds KIEB and DACH (KW; χ^2^ = 17.05, df = 8, *P* = 0.03) differed significantly from each other (Table 3.5, Appendix 3).

### Influence of *chl-a* concentrations on biomass and total lipids of seston and Chironomidae and on n-3 PUFA contents of Chironomidae

**Fig. 3:**
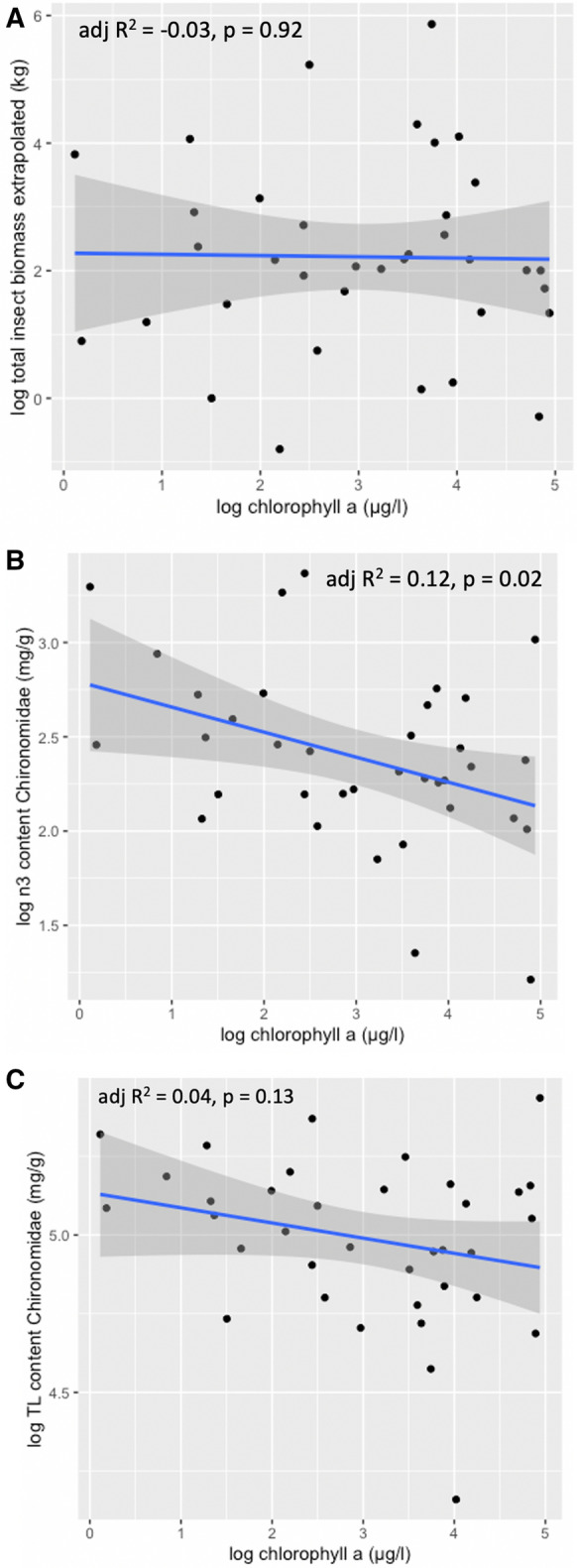
Linear regressions between **A** chlorophyll-a content (µg l^−1^) and extrapolated total insect biomass (kg); **B** n-3 PUFA content of Chironomidae (mg g^−1^), and; **C** total lipids of Chironomidae (mg g^−1^), without taking into account the pond as a random factor. All values were log-transformed

**Fig. 4:**
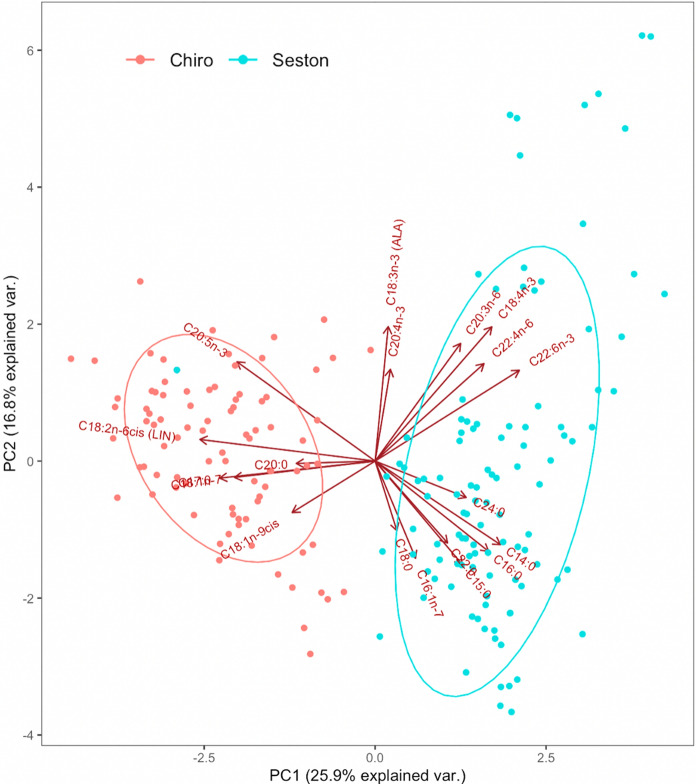
Principal component analysis (PCA) of fatty acids (FA) on Chironomidae and seston (data comprising all of the 9 study ponds). Ellipses indicate 95% confidence intervals

**Fig. 5:**
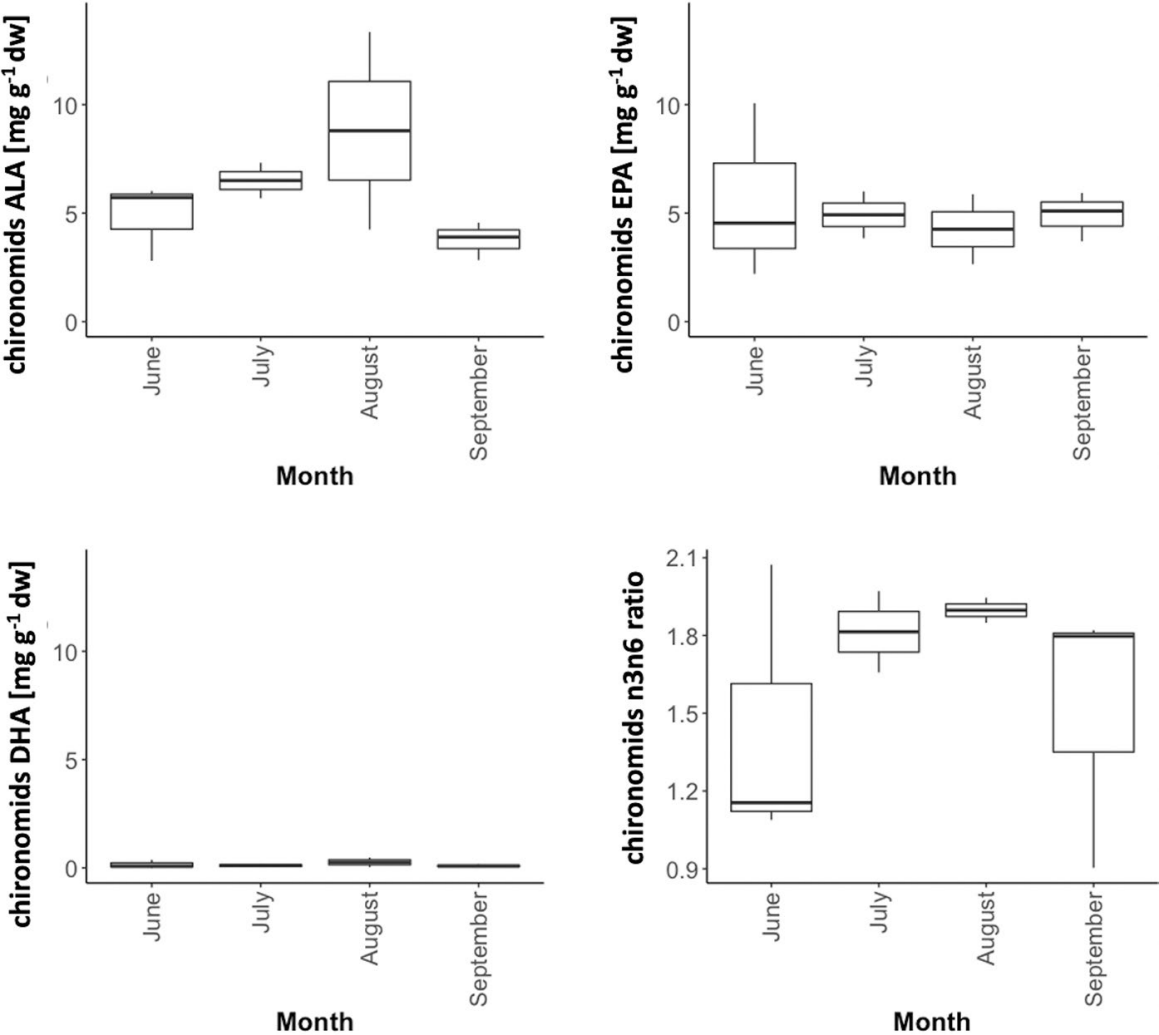
The omega-3 PUFA DHA, EPA, and ALA as well as the n-3:n-6 ratio of Chironomidae during the sampling period in Jägerteich (JAG). Letters indicate significant differences among the months (KW test)

Increasing Chl-*a* concentrations of these fishponds were significantly negatively correlated with the total lipid content in seston, but had no significant effect on seston biomass (Table 1). In contrast, Chl-*a* had a significant negative effect on the exported biomass of Chironomidae and on the total insect biomass (Table 1). In addition, Chl-*a* had a significant negative effect on the total lipids and n-3 PUFA content in Chironomidae (Table 1). Total lipids of seston had a significant negative effect on the total lipids in Chironomidae (Table 1). In the linear models, the negative effect of Chl-*a* on n-3 PUFA content of Chironomidae was observed (lm, adj *R*^2^ = 0.12, *P* = 0.02; Fig. 3B), but was not significant for Chironomidae total lipids or exported insect biomass (lm, adj *R*^2^ = 0.04, *P* = 0.13; Fig. 3A and lm, adj *R*^2^ =  − 0.03, *P* = 0.92; Fig. 3C, Fig. 4, Fig. 5, respectively).

**Table 1:** Results of the LME (linear mixed effect) models. Significant differences are listed in bold (p<0.05)

Model	Model marginal	Terms	Slope estimate, 95% CI	Std. error	df	*t*-values	*P*-values
Effect of Chl-a on seston biomass	*R*^2^ = 0.002	Seston biomass	1.54	0.19	26	8.21	** < 0.001**
		Chl-a	0.02 [− 0.09, 0.13]	0.05	26	0.31	0.76
Effect of Chl-a on seston lipids	*R*^2^ = 0.14	Seston lipids	4.11	0.16	26	24.95	** < 0.001**
		Chl-a	0.13 [0.05, 0.21]	0.04	26	3.27	**0.003**
Effect of Chl-a on Chirono biomass	*R*^2^ = 0.43	Chirono biomass	3.02	0.71	26	4.23	** < 0.001**
		Chl-a	− 0.46 [− 0.66, − 0.26]	0.1	26	− 4.68	** < 0.001**
Effect of Chl-a on insect biomass export	*R*^2^ = 0.34	Insect biomass	3.19	0.57	26	5.58	**<0.001**
		Chl-a	− 0.32 [− 0.50, − 0.15]	0.08	26	− 3.81	** < 0.001**
Effect of seston lipids on Chirono lipids	*R*^2^ = 0.19	Chirono Lipids	4.83	2.38	26	2.02	0.05
		Seston Lipids	− 1.14 [− 2.2, − 0.1]	0.52	26	− 2.2	**0.04**
Effect of Chl-a on Chirono lipids	*R*^2^ = 0.51	Chirono Lipids	1.28	0.7	26	1.81	0.08
		Chl-a	− 0.52 [− 0.72, − 0.32]	0.1	26	− 5.46	** < 0.001**
Effect of Chl-a on n-3 PUFA of Chirono	*R*^2^ = 0.43	Chirono n-3 PUFA	− 1.14	0.77	26	− 1.49	0.15
		Chl-a	− 0.57 [− 0.82, − 0.32]	0.12	26	− 4.67	** < 0.0001**

Terms describe the fixed effects used in the model, with the random effect being always “pond.” *Chirono* Chironomidae, *Std.Error* standard error, *Df* degrees of freedom, *Chl-a* chlorophyll-a content [µg l^−1^], *Model Marginal R*^2^ variance explained by fixed effects, *Slope estimate* estimate value of deviation from the slope, 95% Confidence Intervals (CI) for the independent variables are reported. LME values were log-transformed prior to the analysis

## Discussion

Emergent insects from these nine eutrophic fishponds supplied 1068 kg of total insect biomass, of which 110 kg were total lipids and > 10 kg n-3 PUFA, from aquatic to terrestrial ecosystems throughout the 4 month study period. Emergent biomass, total lipids, and n-3 PUFA contents of Chironomidae decreased with increasing Chl-*a* concentrations, indicating that biomass and dietary nutrient mobilization via this taxon from fishponds to terrestrial ecosystems was sensitive to the trophic condition of ponds. The export of lipids and n-3 PUFA in this particular study was primarily from Chironomidae which had the highest biomass exported, making this taxonomic group of emergent insects an important source of dietary energy and, in particular, n-3 PUFA for consumers in the terrestrial ecosystems adjacent to fishponds. The total lipid and FA content of seston varied among months and ponds, but did not significantly affect the total lipid and n-3 PUFA content of Chironomidae. Similarly, there was no significant difference in the n-3 PUFA (i.e., DHA, EPA, and ALA) content of Chironomidae among ponds, suggesting that emergent insects were able to selectively retain these lipids and/or potentially biosynthesize these important n-3 PUFA.

During the four months of sampling, the continuous addition of fish feed (pers. comm. with the fish farmers) likely contributed to rising nutrient supply that translated to increasing Chl-*a* concentrations in the fishponds (Hlaváč et al., 2014). In DURN, which was not used for fish-farming and not subject to feed supply, the Chl-*a* concentrations decreased in September after reaching its peak in August, suggesting high grazing pressure on algae and/or seasonal algal biomass fluctuations as reported from other shallow lakes (Padisák, 1993). Despite the seasonal increase of Chl-*a* concentrations, the overall emergence of insect biomass did not significantly vary among months, indicating a steady supply of dietary energy to the terrestrial environment throughout the study period. However, contrary to our hypothesis, we found a negative relationship between the biomass of emergent Chironomidae and Chl-*a* concentrations, suggesting that seasonal changes in pond trophic conditions were not associated with alterations in longer term insect emergence events. In addition, since we had high variation in Chl-*a* values (i.e., pond productivity) among the ponds, this may have masked the effect of time (months).

Greig et al. (2012) found a positive nutrient-mediated bottom-up control of insect emergence in an outdoor mesocosm experiment. In the ponds we studied here, however, the overall high nutrient concentrations may have already exceeded the level beneficial for insect emergence and may have even become a hindrance to the emergence of Chironomidae during the summer, as suggested by Scharnweber et al. (2019). Increasing eutrophication also increases turbidity and, as a consequence, decreases light penetration to the sediment–water interface, which has been suggested to be a strong driver decreasing insect emergence (Ivković et al., 2013). In addition to bottom-up driven regulations, the emergence of pond insects can also be regulated by feeding pressure of fishes (here carp), as recently demonstrated in a mesocosm study (Scharnweber et al., 2019; but see Greig et al., 2012). Additionally, in more eutrophic ponds like ours, water temperatures are often high and dissolved oxygen is reduced, resulting in fewer sensitive taxa such as Odonata or Trichoptera (Lewis-Phillips et al., 2020) and leading to a bias in the sampling toward generalist taxa like Chironomidae and Chaoboridae. More diverse insect communities might benefit terrestrial consumers differently, by providing more diverse FA contributions to their diet (Mathieu-Resuge et al. 2021b).

The main differences in emergent insect biomass in the sampled ponds in this study were between DURN and FURT, and the ponds GER, HERR, and STADT, all of which contained quite different fish communities and thus might be subject to variations in the predation pressure. The pond JAG was also different in its biomass export from the other ponds, but it was also the biggest pond sampled compared to the others. Looking at emergence values per square meter, the ponds did not differ from each other when considering total biomass of emergent insects or Chironomidae. Potential reasons besides fish predation which could account for differences in insect emergence, especially regarding the biodiversity, might be pond connectivity, since the ponds ENG, GER, and HERR were more isolated than the five ponds close to Waidhofen an der Thaya. It is well known that pond connectivity enhances diversity, including macroinvertebrates (Florencio et al., 2013).

Although in the present study we did not examine the potential top-down feeding pressure by fish or connectivity to other water bodies, the steady export of insects emerged from these ponds indicates continuous availability of emergent insects throughout the spring and summer months for terrestrial consumers, such as insectivorous birds (Twining et al., 2018) and spiders (Mathieu-Resuge et al., 2021b). This suggests that fishponds provide a considerable amount of dietary nutrients to riparian consumers during the summer months.

Shallow ponds are more easily mixed than lakes (Andersen et al., 2017) and thus pelagic algae-derived PUFA are likely available to insect larvae at the sediment–water interface. Although not specifically tested in this study, the difference of PUFA composition among insect taxa might be partly attributed to the fact that many of the emergent insect species are bivoltine or multivoltine and thus acquire their PUFA from different phytoplankton communities or biofilms at the sediment–water interface during their larval stage (Iverson, 2009; Guo et al., 2021). In addition, insects that have longer larval cycles, in some species lasting longer than one year, can integrate nutrients from more than just one season (Cayrou & Céréghino, 2005). Insect larvae may retain dietary PUFA selectively and thus their dietary PUFA composition may not predict the larval or imago PUFA composition of insects, as reported for insect larvae in streams (Guo et al., 2018). Gladyshev et al. (2009) reported similar FA contents between adult Chironomidae and their larval stages, suggesting that the PUFA composition of the adult emergent insects was similar to preceding conditions. The potential of insect larvae to selectively retain n-3 PUFA might be physiologically important in these eutrophic ecosystems that generally contain high cyanobacteria biomass and thus potentially provide low levels of dietary LC-PUFA (Martin-Creuzburg et al., 2008). Strandberg et al. (2020) reported that Chironomidae (*Chironomus riparius*, Johann Wilhelm Meigen 1804) selectively synthesize EPA from precursor molecules when feeding on certain cyanobacteria that contain ALA. In view of the usually rather short life span of adult Chironomidae, in which they do not feed at all or only on “flight fuel” (e.g., nectar; Oliver, 1971), it is plausible that this taxon developed ways to retain PUFA for their physiological needs prior to their emergence. Chironomidae may thus not merely be seen as ‘collectors’ of dietary PUFA, but as key consumers that are able to also convert dietary PUFA to LC-PUFA.

The changing taxonomic assemblages of emergent Chironomidae and Chaoboridae throughout the summer in the study ponds indicate a temporarily heterogeneous availability of insects and thus LC-PUFA for riparian consumers. This is most evident in systems with a high abundance of Chaoboridae, which contribute most to the total DHA export (Martin-Creuzburg et al., 2017). Although it is not known which trophic and/or environmental conditions favor the emergence of DHA-rich Chaoboridae from fishponds, it is clear that emergent insects generally supply nutritionally higher food quality than that provided to them by algae of these eutrophic ecosystems. Thus, the biodiversity of emerged insects from ponds is crucial for the provision of dietary PUFA diversity to terrestrial consumers.

This study shows that fishponds, which have mostly been looked at from a fish perspective (e.g., Chumchal et al., 2005), provide valuable dietary PUFA from emergent insects to riparian consumers. The eutrophic fishponds (total surface ~ 68 ha) yielded a total of 1068 kg of emergent insects over four summer months, of which Chironomids released 9 kg n-3 PUFA to adjacent ecosystems, while a subalpine oligotrophic lake of the same size, sampled for the same amount of time a year earlier, yielded 462 kg of emergent insects, of which Chironomids transferred 4 kg n-3 PUFA (Mathieu-Resuge et al., 2021b). This difference indicates that Chironomids from eutrophic ponds may release higher n-3 PUFA contents per area than oligotrophic lakes, which contrasts earlier findings that increasing trophic lake status decreases dietary supply of n-3 PUFA (Müller-Navarra et al., 2004). The distinct difference between low contents of dietary PUFA in seston and high n-3 LC-PUFA values in emerged insects demonstrates that emergent insects convert and/or selectively retain n-3 LC-PUFA from their diet sources. The high n-3 LC-PUFA contents found in emergent insects make them crucial for supplying these important nutrients from eutrophic ponds to subsequent consumers. Importantly, the high amounts of nutrients provided by aquatic insects emerging from ponds may also be crucial for supporting biodiversity of riparian consumers.

In conclusion, our study underlines the so far underestimated ecological values of fishponds across ecosystems. In our eutrophic study fishponds, the emergent insect biomass per square meter was lower than from other managed farm ponds (Lewis-Phillips et al., 2020), possibly because of higher feeding pressure of insectivorous fish that may have also altered the taxonomic composition of emergent insects. Finally, this study reveals that man-made ponds, even if used as managed fishponds, provide as yet largely unrecognized ecosystem services from aquatic to terrestrial ecosystems via the tremendous export of emergent insects.

## Supplementary Information

Below is the link to the electronic supplementary material.

Supplementary file1 (DOCX 124 kb)

Supplementary file2 (PNG 23575 kb)

## Data Availability

Data will be made available upon reasonable request.
