# Supplementary material for: Export of dietary lipids via emergent insects from eutrophic fishponds
Source: Hydrobiologia. 2022 Oct 27;850(15):3241–56. doi: 10.1007/s10750-022-05040-2 (PMC10307721; doi:10.1007/s10750-022-05040-2)
Supplement: Supplementary file 1 — Supplementary file1 (DOCX 124 kb) [file 10750_2022_5040_MOESM1_ESM.docx]

**Appendix 1 - Map of field sampling site**

Figure 1.1:

(A) Map of Austria, established with leaflet package in R (data from OpenStreetMap). Selection of the region of Austria where the Waldviertel region is located (black square selection, manually selected). (B) Overview of all pond sites (blue circles, *addCircleMarkers* in *leaflet)*. In the upper part, the area around the village Kautzen with the two ponds ENG and GER. In the middle part, the area around Waidhofen an der Thaya with the ponds JAG, KIEB, FURT, DACH, STADT close to each other and HERR a bit more outside. In the lower part, the pond DURN in the area of Zwettl. Red arrow signs point to the geographical North.

**Appendix 2 - Physiochemical parameters, pond surface sizes and seston biomass values**

**Suppl. Table 2.1**:

Physical and chemical parameters of the ponds throughout the sampling period. DOC (mean ± SD). NA = no measurements (i.e., in July, no multiparameter-sonde measurements were taken due to logistical problems). Fish ponds: JAG=Jägerteich, KIEB=Kiebitzteich, FURT=Furtteich, DACH=Dachetteich, STADT=Stadtteich, HERR=Herrenteich, ENG=Engelbrechtsteich, GER= Gerhartsteich, DURN=Dürnhofteich.

| Pond | Month | Pond temperature (˚C) | pH | Conductivity (mS/cm) | Chl-*a* [µg / l] | DOC (mean ± SD) [mg/l] |
| --- | --- | --- | --- | --- | --- | --- |
| JAG | June | 17.4 | 8.3 | 0.4 | 12.2 | 11.5 ± 0.3 |
|  | July | NA | NA | NA | 36.4 | 13.1 ± 0.2 |
|  | August | 26.7 | 8.6 | 0.3 | 43.5 | 12.8 ± 0.1 |
|  | September | 18.4 | 7.7 | 0.3 | 42.3 | 14.7 ± 0.2 |
| KIEB | June | 18.0 | 7.5 | 0.6 | 1.1 | 8.3 ± 0.1 |
|  | July | NA | NA | NA | 5.3 | 9 ± 0.3 |
|  | August | 24.2 | 7.7 | 0.5 | 133.3 | 8.5 ± 0.3 |
|  | September | 17.8 | 7.4 | 0.5 | 139.9 | 8.8 ± 0.7 |
| FURT | June | 16.8 | 7.5 | 0.2 | 2.3 | 15.6 ± 0.3 |
|  | July | NA | NA | NA | 52.4 | 16.1 ± 0.1 |
|  | August | 22.7 | 7.5 | 0.2 | 38 | 13.2 ± 1.5 |
|  | September | 16.3 | 7.4 | 0.2 | 125.9 | 11.3 ± 0.3 |
| DACH | June | 17.7 | 7.5 | 0.3 | 3.9 | 8.6 ± 0.1 |
|  | July | NA | NA | NA | 8.6 | 9.8 ± 0.1 |
|  | August | 24.3 | 7.5 | 0.2 | 25.3 | 10.5 ± 0.3 |
|  | September | 17.8 | 7.1 | 0.2 | 69.9 | 9.4 ± 0.2 |
| STADT | June | 17.2 | 8.4 | 0.2 | 3.6 | 11.9 ± 0.1 |
|  | July | NA | NA | NA | 33.4 | 13 ± 1.7 |
|  | August | 26.0 | 8.8 | 0.2 | 111 | 14.8 ± 0.4 |
|  | September | 17.0 | 7.3 | 0.2 | 128 | 15.8 ± 0.8 |
| HERR | June | 17.1 | 8.1 | 0.2 | 7.3 | 10.3 ± 0.6 |
|  | July | NA | NA | NA | 49 | 10.5 ± 0.4 |
|  | August | 24.3 | 9.3 | 0.2 | 48.1 | 12.3 ± 0.5 |
|  | September | 16.9 | 8.5 | 0.2 | 55.7 | 10.9 ± 0.4 |
| ENG | June | 16.2 | 7.1 | 0.1 | 11.5 | 8.7 ± 0.2 |
|  | July | NA | NA | NA | 19.5 | 9.6 ± 0.0 |
|  | August | 24.4 | 8.2 | 0.1 | 17.4 | 9.4 ± 0.5 |
|  | September | 17.6 | 8.1 | 0.1 | 11.5 | 8.2 ± 0.2 |
| GER | June | 16.8 | 8.0 | 0.2 | 3.8 | 13.6 ± 0.1 |
|  | July | NA | NA | NA | 31.9 | 8.1 ± 0.6 |
|  | August | 25.2 | 9.4 | 0.2 | 62.1 | 9.4 ± 0.3 |
|  | September | 17.5 | 8.5 | 0.2 | 65.8 | 8.7 ± 0.1 |
| DURN | June | 16.4 | 7.0 | 0.7 | 1.2 | 10.3 ± 0.2 |
|  | July | NA | NA | NA | 4.5 | 10.6 ± 0.1 |
|  | August | 22.7 | 7.1 | 0.6 | 13.2 | 11.6 ± 0.6 |
|  | September | 16.5 | 6.8 | 0.7 | 9 | 12.9 ± 0.7 |

**Appendix 3 - Extrapolated insect biomass values and n-3 PUFA and total lipids values**

**Suppl. Table 3.1:**

Biomass (kg dw extrapolated per pond area) of all emerged insects and specifically of Chironomidae, Chaoboridae and Odonata from each of the study ponds. Pond surface area in hectares (ha). Fish ponds: JAG=Jägerteich, KIEB=Kiebitzteich, FURT=Furtteich, DACH=Dachetteich, STADT=Stadtteich, HERR=Herrenteich, ENG=Engelbrechtsteich, GER=Gerhartsteich, DURN=Dürnhofteich.

| Pond | Month | Total insects (kg) | Chironomidae (kg) | Chaoboridae (kg) | Odonata (kg) | Pond surface size (ha) |
| --- | --- | --- | --- | --- | --- | --- |
| JAG | June | 186.4 | 112.5 | 0.4 | 0 | 38.1 |
|  | July | 73.4 | 50.3 | 10.2 | 0 | 0 |
|  | August | 55.0 | 39.7 | 13.5 | 0 | 0 |
|  | September | 352.8 | 279.3 | 48.0 | 0 | 0 |
| KIEB | June | 45.8 | 31.9 | 2.4 | 3.8 | 2.5 |
|  | July | 4.4 | 2.6 | 1.3 | 0 | 0 |
|  | August | 5.6 | 1.7 | 3.2 | 0 | 0 |
|  | September | 3.8 | 1.6 | 2.1 | 0 | 0 |
| FURT | June | 3.3 | 2.2 | 0.5 | 0.1 | 0.7 |
|  | July | 1.3 | 0.2 | 0.8 | 0 | 0 |
|  | August | 1.2 | 0.2 | 0.9 | 0 | 0 |
|  | September | 0.8 | 0.2 | 0.5 | 0 | 0 |
| DACH | June | 10.8 | 7.1 | 0.3 | 0 | 3.0 |
|  | July | 8.8 | 3.0 | 2.2 | 0 | 0 |
|  | August | 7.6 | 4.1 | 2.0 | 0 | 0 |
|  | September | 3.9 | 2.0 | 1.5 | 0 | 0 |
| STADT | June | 58.3 | 47.4 | 0 | 0 | 3.9 |
|  | July | 9.6 | 6.7 | 0.5 | 0 | 0 |
|  | August | 7.4 | 3.5 | 1.7 | 1.1 | 0 |
|  | September | 7.4 | 3.9 | 3.0 | 0.1 | 0 |
| HERR | June | 23.0 | 16.6 | 0.1 | 0 | 10.1 |
|  | July | 17.6 | 12.6 | 0.4 | 0 | 0 |
|  | August | 13.0 | 10.9 | 0.6 | 0 | 0 |
|  | September | 60.5 | 57.4 | 1.8 | 0 | 0 |
| GER | June | 18.5 | 14.5 | 0.0 | 0 | 3.4 |
|  | July | 8.9 | 7.9 | 0.1 | 0 | 0 |
|  | August | 8.8 | 7.7 | 0.1 | 0 | 0 |
|  | September | 29.4 | 28.9 | 0 | 0 | 0 |
| ENG | June | 6.8 | 6.1 | 0 | 0 | 3.0 |
|  | July | 7.9 | 7.4 | 0.1 | 0 | 0 |
|  | August | 5.4 | 4.7 | 0.2 | 0 | 0 |
|  | September | 15.1 | 14.5 | 0.4 | 0 | 0 |
| DURN | June | 2.5 | 1.7 | 0.5 | 0 | 0.6 |
|  | July | 1.0 | 0.2 | 0.7 | 0 | 0 |
|  | August | 2.1 | 0.3 | 1.6 | 0 | 0 |
|  | September | 0.5 | 0.2 | 0.2 | 0 | 0 |

**Suppl. Table 3.2:**

Biomass (mg dw, averages per square meter) of all emerged aquatic insects from each of the study ponds. Fish ponds: JAG=Jägerteich, KIEB=Kiebitzteich, FURT=Furtteich, DACH=Dachetteich, STADT=Stadtteich, HERR=Herrenteich, ENG=Engelbrechtsteich, GER=Gerhartsteich, DURN=Dürnhofteich. Chirono=Chironomidae; Chao=Chaoboridae; Tricho=Trichoptera; Ephemero=Ephemeroptera; Odo=Odonata

| **Pond** | **Month** | **Total biomass per m^2^ (mg)** | **Chirono biomass per m^2^ (mg)** | **Chao biomass per m^2^ (mg)** | **Tricho biomass per m^2^ (mg)** | **Ephemero biomass**  **per m^2^ (mg)** | **Odo biomass per m^2^ (mg)** |
| --- | --- | --- | --- | --- | --- | --- | --- |
| JAG | June | 81.82 | 48.52 | 0.34 | 3.01 | 0.0 | 0.0 |
|  | July | 59.29 | 37.45 | 5.65 | 0.0 | 2.05 | 0.0 |
|  | August | 93.21 | 66.71 | 23.68 | 0.59 | 0.0 | 0.0 |
|  | September | 139.54 | 111.26 | 18.41 | 1.26 | 0.0 | 0.0 |
| KIEB | June | 274.04 | 166.85 | 27.76 | 0.0 | 5.72 | 30.70 |
|  | July | 83.96 | 50.57 | 21.66 | 0.62 | 0.50 | 0.0 |
|  | August | 36.55 | 5.86 | 26.88 | 0.56 | 1.76 | 0.0 |
|  | September | 106.94 | 39.48 | 55.59 | 1.89 | 3.43 | 0.0 |
| FURT | June | 90.45 | 51.72 | 19.09 | 1.23 | 0.0 | 5.21 |
|  | July | 85.46 | 11.72 | 48.49 | 2.78 | 8.52 | 0.0 |
|  | August | 58.35 | 10.64 | 47.26 | 0.0 | 0.0 | 0.0 |
|  | September | 49.65 | 7.81 | 40.49 | 0.0 | 1.34 | 0.0 |
| DACH | June | 61.64 | 38.9 | 2.27 | 1.0 | 0.0 | 0.0 |
|  | July | 141.99 | 44.91 | 34.74 | 11.0 | 37.32 | 0.0 |
|  | August | 46.29 | 29.4 | 13.78 | 2.38 | 0.60 | 0.0 |
|  | September | 101.16 | 49.23 | 26.54 | 13.58 | 11.33 | 0.0 |
| STADT | June | 258.82 | 206.35 | 0.0 | 15.23 | 0.0 | 0.0 |
|  | July | 113.6 | 79.5 | 2.19 | 5.51 | 1.88 | 0.0 |
|  | August | 60.39 | 29.82 | 16.07 | 1.63 | 0.50 | 8.34 |
|  | September | 80.06 | 41.36 | 20.84 | 6.05 | 1.11 | 6.87 |
| HERR | June | 39.49 | 24.28 | 0.17 | 3.13 | 0.0 | 0.0 |
|  | July | 59.23 | 35.13 | 1.08 | 4.85 | 2.44 | 0.0 |
|  | August | 79.4 | 71.51 | 2.88 | 2.47 | 2.29 | 0.0 |
|  | September | 94.29 | 86.1 | 3.6 | 3.90 | 0.0 | 0.0 |
| GER | June | 76.62 | 59.2 | 0.0 | 8.05 | 0.0 | 0.0 |
|  | July | 78.77 | 64.76 | 0.83 | 7.43 | 0.0 | 0.0 |
|  | August | 147.84 | 130.02 | 1.02 | 15.73 | 0.0 | 0.0 |
|  | September | 149.53 | 147.46 | 0.0 | 1.21 | 0.0 | 0.0 |
| ENG | June | 33.53 | 30.10 | 0.0 | 0.22 | 0.0 | 0.0 |
|  | July | 108.35 | 101.1 | 0.31 | 2.11 | 1.43 | 0.0 |
|  | August | 95.72 | 85.65 | 2.92 | 6.63 | 0.0 | 0.0 |
|  | September | 71.37 | 67.67 | 1.61 | 0.0 | 1.12 | 0.0 |
| DURN | June | 80.74 | 53.6 | 23.43 | 0.0 | 0.0 | 0.0 |
|  | July | 65.11 | 11.04 | 40.59 | 0.0 | 11.30 | 0.0 |
|  | August | 138.52 | 20.78 | 109.55 | 1.78 | 4.99 | 0.0 |
|  | September | 59.81 | 14.05 | 32.66 | 0.66 | 11.99 | 0.0 |

**Suppl. Table 3.3:**

Extrapolated total lipids and omega-3 PUFA export via Chironomidae, Chaoboridae and Odonata (mg dw per pond area) were calculated in regard to pond surface and the extrapolated Chironomidae biomass. Fish ponds: JAG=Jägerteich, KIEB=Kiebitzteich, FURT=Furtteich, DACH=Dachetteich, STADT=Stadtteich, HERR=Herrenteich, ENG=Engelbrechtsteich, GER=Gerhartsteich, DURN=Dürnhofteich.

| **Pond** | **Month** | **n-3 PUFA Chironomidae (g)** | **n-3 PUFA Chaoboridae (g)** | **n-3 PUFA Odonata (g)** | **Total lipids Chironomidae**  **(g)** | **Total lipids Chaoboridae (g)** | **Total lipids Odonata (g)** |
| --- | --- | --- | --- | --- | --- | --- | --- |
| **JAG** | June | 1269.3 | NA | 0 | 18315.5 | NA | 0 |
|  | July | 616.5 | 391.6 | 0 | 5969.3 | 1325.4 | 0 |
|  | August | 572.1 | 399.4 | 0 | 5589.8 | 2764.3 | 0 |
|  | September | 2727.8 | NA | 0 | 27089.9 | NA | 0 |
| **KIEB** | June | 859.3 | NA | 52.8 | 6506.9 | NA | 414.5 |
|  | July | 34.1 | 35.8 | 0 | 362.3 | 153.1 | 0 |
|  | August | 5.6 | 68.9 | 0 | 181.0 | 377.7 | 0 |
|  | September | 32.4 | NA | 0 | 364.5 | NA | 0 |
| **FURT** | June | 41.9 | NA | 1.7 | 396.5 | NA | 13.1 |
|  | July | 1.6 | 21.3 | 0 | 28.5 | 100.8 | 0 |
|  | August | 0.7 | 26.7 | 0 | 21.6 | 154.8 | 0 |
|  | September | 2.4 | NA | 0 | 38.2 | NA | 0 |
| **DACH** | June | 86.6 | NA | 0 | 1126.7 | NA | 0 |
|  | July | 35.1 | 62.9 | 0 | 449.9 | 286.2 | 0 |
|  | August | 26.2 | 75.7 | 0 | 705.2 | 446.3 | 0 |
|  | September | 20.5 | NA | 0 | 240.4 | NA | 0 |
| **STADT** | June | 722.2 | NA | 0 | 9348.9 | NA | 0 |
|  | July | 46.0 | 8.6 | 0 | 890.1 | 50.5 | 0 |
|  | August | 27.5 | 46.4 | 15.0 | 592.2 | 334.8 | 152.8 |
|  | September | 58.0 | NA | 0.9 | 608.4 | NA | 9.7 |
| **HERR** | June | 254.5 | NA | 0 | 2832.8 | NA | 0 |
|  | July | 120.7 | 6.9 | 0 | 1594.6 | 37.9 | 0 |
|  | August | 170.8 | 17.3 | 0 | 1536.8 | 104.8 | 0 |
|  | September | 479.7 | NA | 0 | 3680.2 | NA | 0 |
| **GER** | June | 114.5 | NA | 0 | 2399.2 | NA | 0 |
|  | July | 79.9 | 1.8 | 0 | 1499.6 | 10.0 | 0 |
|  | August | 87.8 | NA | 0 | 1255.4 | NA | 0 |
|  | September | 431.8 | NA | 0 | 4048.8 | NA | 0 |
| **ENG** | June | 177.5 | NA | 0 | 1314.7 | NA | 0 |
|  | July | 68.3 | NA | 0 | 817.6 | NA | 0 |
|  | August | 42.6 | 4.2 | 0 | 675.5 | 24.7 | 0 |
|  | September | 130.2 | NA | 0 | 1956.3 | NA | 0 |
| **DURN** | June | 20.3 | NA | 0 | 281.6 | NA | 0 |
|  | July | 1.5 | 17.6 | 0 | 18.7 | 71.3 | 0 |
|  | August | 2.5 | 43.5 | 0 | 40.7 | 282.0 | 0 |
|  | September | 6.4 | NA | 0 | 44.0 | NA | 0 |

**Suppl. Table 3.4:**

Seston omega-3 PUFA contents (i.e. DHA, EPA, ALA) and the n-3:n-6 ratios (mean ± SD; mg g dw ^-1^) of the nine sampled ponds throughout the sampling season (June-September 2020). The letters indicate significant differences among the ponds for each fatty acid (KW tests with Conover post-hoc test and Holm-Bonferroni correction), considering all months sampled. Fish ponds: JAG=Jägerteich, KIEB=Kiebitzteich, FURT=Furtteich, DACH=Dachetteich, STADT=Stadtteich, HERR=Herrenteich, ENG=Engelbrechtsteich, GER=Gerhartsteich, DURN=Dürnhofteich.

|  | **JAG** | | | |  | **KIEB** | | | |  | **FURT** | | | |
| --- | --- | --- | --- | --- | --- | --- | --- | --- | --- | --- | --- | --- | --- | --- |
|  | June | July | Aug | Sept |  | June | July | Aug | Sept |  | June | July | Aug | Sept |
| DHA(ab) | 0.1± 0 | 0.1± 0 | 0.1± 0 | 1.4± 0.1 | DHA(b) | 0± 0 | 0± 0 | 0.1± 0.1 | 0.3± 0.1 | DHA  (a) | 0.1± 0 | 0.4± 0.1 | 0.8± 0.3 | 1± 0.1 |
| EPA  (abc) | 0.4± 0.1 | 0.3± 0.1 | 0.3± 0.1 | 2.9± 0.3 | EPA  (c) | 0.2± 0 | 0.2± 0 | 0.1± 0.1 | 0.6± 0.1 | EPA  (ab) | 0.3± 0 | 0.8± 0.1 | 1.1± 0.4 | 0.8± 0.1 |
| ALA  (abc) | 1.4± 0.3 | 1.0± 0.2 | 1.2± 0.3 | 6.0± 0.4 | ALA  (d) | 0.2± 0 | 0.3± 0.1 | 0.4± 0.2 | 1.6± 0.4 | ALA  (abc) | 0.4± 0 | 1.4± 0.1 | 2.8± 0.9 | 2.1± 0.2 |
| n3:n6 | 5.0± 0.3 | 2.5± 0.2 | 2.5± 0.2 | 6.4± 0.3 | n3:n6 | 4.8± 1.2 | 1.4± 0.5 | 2.3± 0.4 | 3.1± 0.1 | n3:n6 | 4.1± 0.6 | 2.2± 0.2 | 2.7± 0.1 | 3.2± 0.4 |
|  |  |  |  |  |  |  |  |  |  |  |  |  |  |  |
|  | **DACH** | | | |  | **STADT** | | | |  | **HERR** | | | |
|  | June | July | Aug | Sept |  | June | July | Aug | Sept |  | June | July | Aug | Sept |
| DHA  (ab) | 0.1± 0 | 0.4± 0.1 | 0.2± 0 | 0.4± 0.1 | DHA  (b) | 0± 0 | 0.2± 0 | 0.1± 0 | 0.1± 0 | DHA  (ab) | 0.1± 0 | 0.2± 0 | 0.2± 0 | 0.6± 0 |
| EPA  (abc) | 0.2± 0.1 | 0.5± 0.1 | 0.4± 0 | 0.5± 0.1 | EPA  (bc) | 0.2± 0 | 0.7± 0.1 | 0.3± 0.1 | 0.3± 0 | EPA  (ab) | 0.2± 0.1 | 0.7± 0.2 | 0.8± 0.1 | 1.7± 0.1 |
| ALA  (cd) | 0.6± 0.2 | 0.4± 0.1 | 0.9± 0.1 | 1.3± 0.4 | ALA  (bcd) | 0.3± 0 | 1.0± 0.2 | 1.0± 0.2 | 1.3± 0.1 | ALA  (ab) | 1.9± 0.5 | 1.1± 0.3 | 1.8± 0.3 | 2.2± 0.2 |
| n3:n6 | 3.5± 0.6 | 3± 0.4 | 2.3± 0.2 | 2.8± 0.2 | n3:n6 | 3.4± 0.1 | 2.8± 0.1 | 2.4± 0.1 | 1.7± 0.1 | n3:n6 | 4.9± 0.4 | 4.0± 0.3 | 2.9± 0.2 | 3.0± 0.2 |
|  |  |  |  |  |  |  |  |  |  |  |  |  |  |  |
|  | **GER** | | | |  | **ENG** | | | |  | **DURN** | | | |
|  | June | July | Aug | Sept |  | June | July | Aug | Sept |  | June | July | Aug | Sept |
| DHA  (a) | 0.1± 0 | 0.3± 0.2 | 0.4± 0.1 | 1.2± 0.4 | DHA  (a) | 0.7± 0.1 | 0.2± 0.1 | 0.1± 0 | 0.4± 0.1 | DHA  (a) | 0.1± 0 | 0.6± 0.1 | 0.4± 0.1 | 1.1± 0.1 |
| EPA  (a) | 0.3± 0 | 1.1± 0.5 | 0.8± 0.2 | 2.6± 0.7 | EPA  (a) | 1.6± 0.2 | 0.7± 0.2 | 0.4± 0 | 0.8± 0.1 | EPA  (a) | 0.5± 0.1 | 1.4± 0.2 | 0.5± 0.4 | 2.2± 0.2 |
| ALA  (a) | 1.5± 0.2 | 2.3± 1.0 | 6.0± 1.9 | 5.5± 1.4 | ALA  (a) | 3.3± 0.5 | 3.1± 0.7 | 2.4± 0.3 | 2.6± 0.2 | ALA  (a) | 0.7± 0 | 2.9± 0.2 | 1.8± 0.5 | 6.1± 0.2 |
| n3:n6 | 5.9± 0.1 | 3.0± 0 | 3.3± 0.2 | 4.8± 0.9 | n3:n6 | 25.7± 7.9 | 2.2± 0.1 | 2.1± 0.3 | 3.0± 0.7 | n3:n6 | 2.8± 0.3 | 5.9± 0.3 | 1.6± 0.9 | 6.2± 0.5 |

**Suppl. Table 3.5**:

Chironomid omega-3 PUFA contents (i.e. DHA, EPA, ALA) and n-3:n-6 ratios (mean ± SD; mg g dw ^-1^) of the study ponds throughout the sampling season (June-September 2020). The letters indicate significant differences among the ponds for each fatty acid (KW tests with Conover post-hoc test and Holm-Bonferroni correction), considering all months sampled. Fish ponds: JAG=Jägerteich, KIEB=Kiebitzteich, FURT=Furtteich, DACH=Dachetteich, STADT=Stadtteich, HERR=Herrenteich, ENG=Engelbrechtsteich, GER=Gerhartsteich, DURN=Dürnhofteich.

|  | **JAG** | | | |  | **KIEB** | | | |  | **FURT** | | | |
| --- | --- | --- | --- | --- | --- | --- | --- | --- | --- | --- | --- | --- | --- | --- |
|  | June | July | Aug | Sept |  | June | July | Aug | Sept |  | June | July | Aug | Sept |
| DHA  (ab) | 0.2± 0.2 | 0.1± 0.1 | 0.3± 0.3 | 0.1± 0.1 | DHA  (a) | 1.0± 1.7 | 0± 0 | 0± 0 | 0± 0 | DHA  (ab) | 0.1± 0.1 | 0.1± 0 | 0± 0 | 0.1± 0 |
| EPA | 5.6± 4.0 | 4.9± 1.5 | 4.3± 2.3 | 4.9± 1.1 | EPA | 9.7± 4.8 | 5.3 ± 1.3 | 0.5± 0 | 4.2± 0.1 | EPA | 6.8± 3.3 | 3.8± 3.1 | 1.4± 0 | 3.8± 0 |
| ALA | 4.9± 1.8 | 6.5± 1.2 | 8.8± 6.4 | 3.8± 0.9 | ALA | 15.1± 9.7 | 7.6± 1.2 | 2.4± 0 | 15.7± 5.2 | ALA | 11.2± 4.7 | 5.3± 4.2 | 2.0± 0 | 5.8± 0 |
| n3:n6 | 1.4± 0.6 | 1.8± 0.2 | 1.9± 0.1 | 1.5± 0.5 | n3:n6 | 3.0± 0.6 | 1.2± 0.2 | 1.4± 0 | 1.7± 0.5 | n3:n6 | 1.7± 0.7 | 1.3± 0.3 | 0.9± 0 | 1.2± 0 |
|  |  |  |  |  |  |  |  |  |  |  |  |  |  |  |
|  | **DACH** | | | |  | **STADT** | | | |  | **HERR** | | | |
|  | June | July | Aug | Sept |  | June | July | Aug | Sept |  | June | July | Aug | Sept |
| DHA  (b) | 0.6± 0.5 | 0.1± 0.1 | 0.1± 0.1 | 0.5± 0 | DHA  (ab) | 0.1± 0.1 | 0.1± 0.1 | 0.2± 0.2 | 0± 0 | DHA  (ab) | 0.1± 0 | 0.1± 0.1 | 0± 0 | 0± 0 |
| EPA | 6.3± 2.9 | 5.7± 1.5 | 2.7± 0.2 | 5.0± 2.8 | EPA | 8.3± 5.1 | 3.1± 1.2 | 4.2± 2.1 | 7.3± 0 | EPA | 9.2± 3.6 | 4.2± 2.1 | 5.8± 2.3 | 4.2± 1.0 |
| ALA | 4.6± 2.0 | 4.5± 2.4 | 3.1± 0.4 | 3.9± 2.2 | ALA | 5.6± 2.2 | 3.3± 1.1 | 3.1± 1.5 | 6.6± 0 | ALA | 5.2± 0.6 | 4.8± 1.6 | 8.5± 4.7 | 3.4± 0.4 |
| n3:n6 | 2.0± 0.9 | 1.1± 0.7 | 1.2± 0.1 | 1.6± 0.1 | n3:n6 | 2.3± 0.8 | 1.1± 0.1 | 0.8± 0.2 | 1.4± 0 | n3:n6 | 1.5± 0.6 | 1.8± 0 | 1.9± 0.4 | 1.7± 0.2 |
|  |  |  |  |  |  |  |  |  |  |  |  |  |  |  |
|  | **GER** | | | |  | **ENG** | | | |  | **DURN** | | | |
|  | June | July | Aug | Sept |  | June | July | Aug | Sept |  | June | July | Aug | Sept |
| DHA  (ab) | 0.3± 0.3 | 0 ± 0 | 0± 0 | 0.1± 0.1 | DHA  (ab) | 0.1± 0.1 | 0± 0 | 0± 0 | 0.1± 0 | DHA  (ab) | 1.3± 0.7 | 0± 0 | 0± 0 | 0± 0 |
| EPA | 4.8± 0.3 | 3.9± 0.8 | 4.4± 2.1 | 5.7± 2.3 | EPA | 10.5± 1.4 | 4.4± 0.9 | 3.0± 2.1 | 4.5± 0.3 | EPA | 4.6± 3.0 | 3.5± 0 | 1.6± 1.5 | 4.1± 0.9 |
| ALA | 2.1± 0.1 | 5.3± 0.3 | 6.3± 1.2 | 8.0± 2.4 | ALA | 15.0± 12.0 | 4.0± 2.5 | 5.33 ± 3.49 | 3.8± 0.3 | ALA | 5.5± 3.3 | 5.3± 0 | 4.6± 3.0 | 16.0± 2.6 |
| n3:n6 | 0.8± 0.3 | 0.9± 0.2 | 1.4± 0.2 | 2.1± 0.4 | n3:n6 | 3.0± 1.9 | 1.4± 0.7 | 1.0± 0.6 | 1.0± 0.1 | n3:n6 | 0.9± 0.4 | 0.8± 0 | 1.3± 0.5 | 2.2± 0 |
