# Supplementary figures and images for: Export of dietary lipids via emergent insects from eutrophic fishponds
Source: Hydrobiologia. 2022 Oct 27;850(15):3241–56. doi: 10.1007/s10750-022-05040-2 (PMC10307721; doi:10.1007/s10750-022-05040-2)

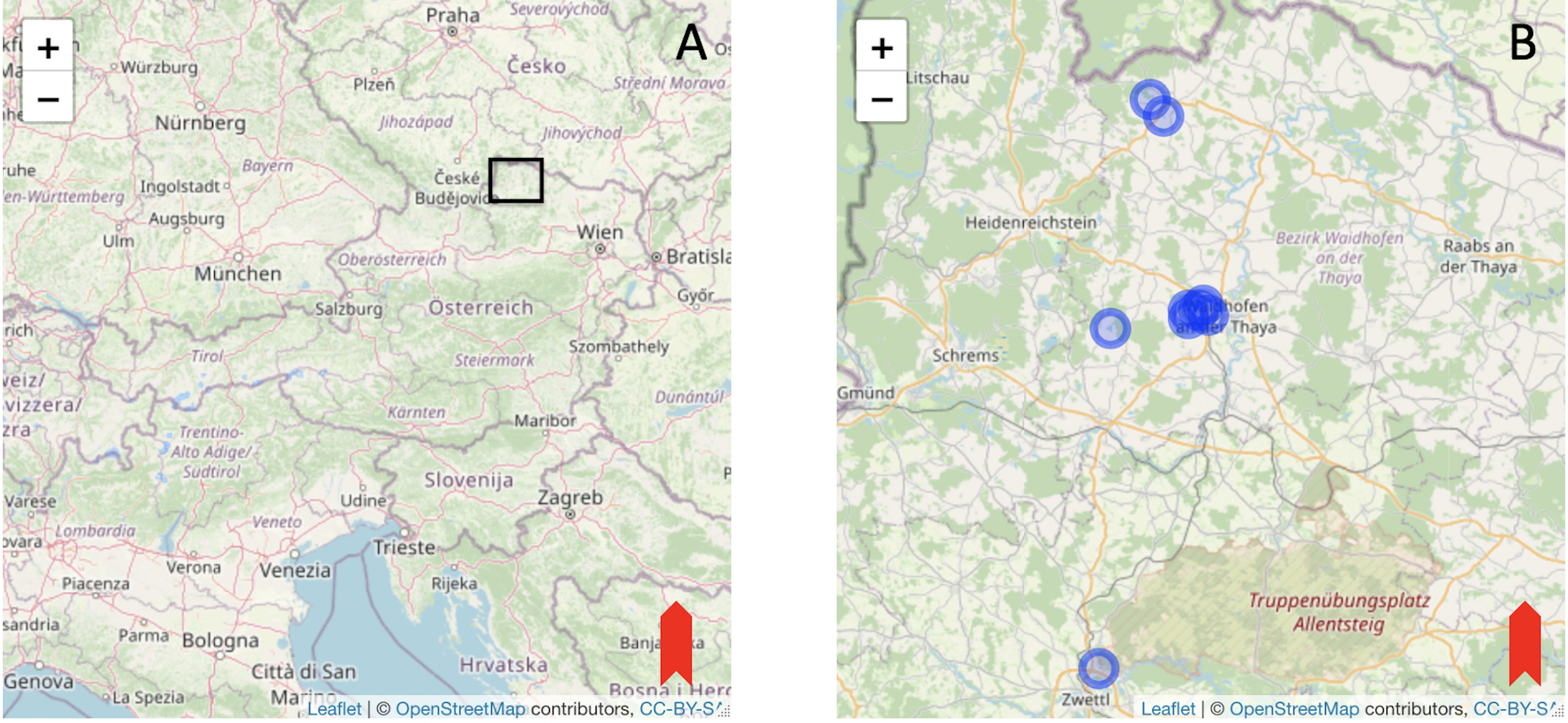

Supplement: Supplementary file 2 — Supplementary file2 (PNG 23575 kb) [file 10750_2022_5040_MOESM2_ESM.png]
